# Effects of Clonidine Premedication Upon Postoperative Shivering and Recovery Time in Patients With and Without Opium Addiction After Elective Leg Fracture Surgeries

**DOI:** 10.5812/aapm.7143

**Published:** 2013-01-01

**Authors:** Morteza Jabbary Moghaddam, Davood Ommi, Alireza Mirkheshti, Ali Dabbagh, Elham Memary, Afsaneh Sadeghi, Mehdi Yaseri

**Affiliations:** 1 Anesthesiology Research Center, Shahid Beheshti University of Medical Sciences, Tehran, Iran; 2 Department of Epidemiology and Biostatistics, Tehran University of Medical Sciences, Tehran, Iran

**Keywords:** Clonidine, Opium, Behavior, Addictive, Shivering

## Abstract

**Background:**

Opium is a highly addictive agent and the most common narcotic often misused in Iran. The pharmacokinetic of anesthetic drugs in patients with opium addiction is one of the great challenges for anesthesiologists. Hemodynamic instability and postoperative side effects are of these challenges which should be managed correctly.

**Objectives:**

In this study we aimed to assess the effects of clonidine upon post anesthesia shivering and recovery time in patients with and without opium addiction after general anesthesia to decrease the subsequent complications related to the shivering and elongation of recovery time.

**Patients and Methods:**

In a randomized clinical trial, 160 patients candidates for elective leg fracture operations under general anesthesia were studied in four groups of 40 patients: Group 1 (placebo 1) were patients without addiction who got placebo 90 minutes before the operation. Group 2 (placebo 2) were patients with opium addiction which received placebo as group 1. Group 3 (Clonidine 1) patients without addiction who got clonidine 90 minutes before the operation and group 4 (Clonidine 2) who were opium addicted ones which received clonidine as premedication.

**Results:**

None of the patients with and without addiction in clonidine groups had shivering after the operation but in placebo groups shivering was observed and the difference between clonidine and placebo groups was statistically significant (*P* < 0.01). Recovery time in clonidine groups of patients with and without addiction was less than placebo ones (both *P* < 0.01) which the magnitude of difference was higher in opium addicted than non-addicted patients (*P* = 0.04).

**Conclusions:**

Premedication with clonidine in patients with and without opium addiction can be effective to decrease the incidence of shivering and recovery time after operation.

## 1. Background

Opium addiction is one of the most common addictions in Iran (1). Patients with opium addiction are more prone to hemodynamic instability which leads to increased risk of general anesthesia in this group of patients. Management of anesthesia in patients with opium addiction is one of the great challenges for anesthesiologists (2). Clonidine as an alpha2 adrenergic agonist has been reviewed in different articles to be able to decrease the hemodynamic responses during surgeries (3-6). It has been shown that clonidine owns anesthetic, sympatholytic, sedative and analgesic properties (7, 8). Newly discovered mechanisms of this drug have been remained as active fields of research (9), moreover it has been shown to be a safe and effective adjuvant drug in patients with addiction (10). Tolerance is a common phenomenon in patients with opium addiction who are candidates for surgeries that require sedation or anesthesia. They would require greater amounts of anesthetics per weight than patients without addiction (11) and they may have some side effects related to their addiction after anesthesia (12). Post anesthesia shivering occurs in 40% of patients (13) and is often preceded by core hypothermia and vasoconstriction (14) but not necessarily so (15). It may cause some complications such as an increase in oxygen consumption, carbon dioxide production, lactic acidosis and release of catecholamines (16). Clonidine acts on central thermoregulatory system and prevents post op shivering (17).

## 2. Objectives

In this study, we assessed the effects of premedication with oral clonidine on postoperative shivering and recovery time after general anesthesia in patients with and without opium addiction who had undergone elective fractured leg surgeries to compare the effects of clonidine upon shivering and recovery time in those two groups of patient.

## 3. Patients and Methods

### 3.1. Patient Selection

After obtaining written informed consent from each patient and approval of Institutional review board, 160 patients who were candidates for elective leg fracture surgeries were selected and entered into our randomized clinical trial. To do this we enrolled all the patients with including criteria for non-addicted group. The opium addicted group was selected with the same sex frequency distribution as non-addicted group: as in each group we had 32 males and eight females. The power as 90% and type I error of α = 0.05. 80 patients were opium addicted and the other 80 were non-addicted ones according to their assertions before the surgery. If any patient used at least 4 grams per day of opium (natural or semisynthetic Alkaloids) as oral or inhalational route for more than six months, we put him or her in opium addicted group. After that we divided each 80 patients into two different groups of 40 patients who either received oral clonidine (about 5 µg/kg) or placebo (vitamin C tablet) by the nurse of anesthesia 90 minutes before the operation. We used Stratified Permuted-block randomization (with the length of 4) to assign our samples to the treatment groups. We had four stratified randomization lists to each combination of Sex-Addiction (Addicted Male, Addicted Female, Non-addicted Male and Non-addicted female). The inclusion criteria of our study were as follow; patients age between 18 to 65 years, American Society of Anesthesiologists (ASA) score one or two, systolic blood pressure during 24 hours before surgery between 90 to 140 mmHg, duration time of operation less than three hours, no history of cardiac diseases or any arrhythmia before surgery, any other fractures or multiple trauma and saturation of Oxygen more than 90% in room air.

### 3.2. Methods

All patients received fentanyl 2 µg/kg intravenously (IV) and midazolam 0.02 mg/kg IV as premedication and induction of anesthesia performed by thiopental 5 mg/kg and Atracurium 0.5 mg/kg IV. For the maintenance of anesthesia we used O_2_/N_2_O as 1/1 liter and propofol by the infusion dosage of 100 µg/kg/min to preserve the value of Cerebral State Index (CSI) between 40 to 60. Systolic and diastolic blood pressure, heart rate, saturation of oxygen and cardiac rhythm monitored and recorded. After the end of the operation and by acquiring the criteria for reversal of neuromuscular blockade, the patients’ endotracheal tubes were extracted and by precise monitoring of hemodynamic and ventilation, they transferred to post anesthesia care unit. In the recovery room the incidence of muscular activity in one or more muscle groups was considered as shivering and treated by Meperidine 20 mg IV. When patients acquired post anesthesia recovery score (modified Aldrete score) > 9, the nurse of anesthesia recorded the time and let them leave the recovery room and transfer to orthopedic ward. The nurse who recorded the incidence of shivering and recovery time of patients did not have any information about opium addiction and premedication of patients. All data were analyzed using SPSS (version 17.0; SPSS Inc, Chicago, IL). For statistical data analysis, student t test, Chi-square test and analysis of variance (ANOVA) with interaction were used. *P* values less than 0.05 were considered significant.

## 4. Results

**Figure 1.**
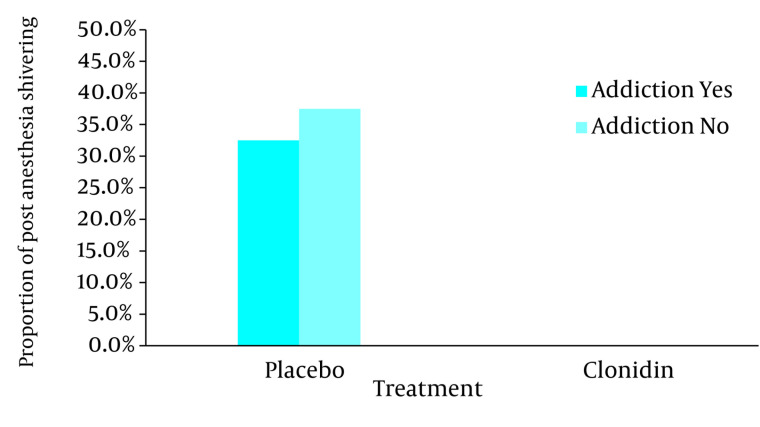
Post Anesthesia Shivering

**Figure 2.**
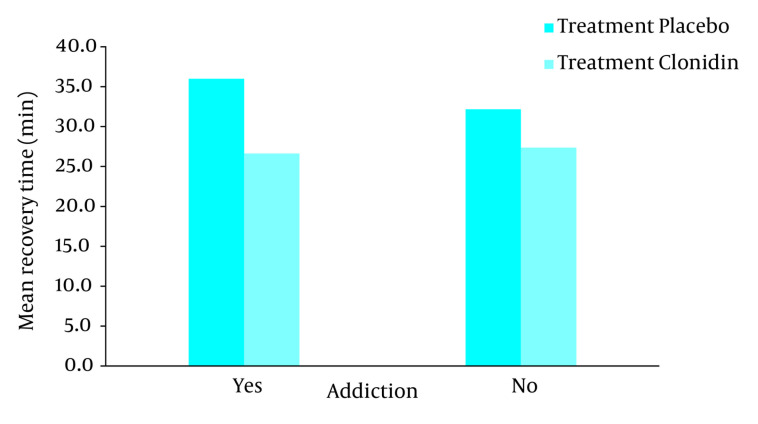
Recovery Time After Operation

In this survey there were no significant differences between all four groups regarding age, weight and duration of operation (Table 1). Post anesthesia shivering was not seen in patients with and without opium addiction who received clonidine as premedication, but 15 patients with opium addiction (37.5%) and 13 patients without opium addiction who received placebo (32.5%) showed post anesthesia shivering (Figure 1). As a result there was a statistical significant difference between clonidine and placebo groups of both patients with and without opium addiction (*P* < 0.01, based on chi-square test) but the difference between opium addicted and non-addicted groups was not statistically different (*P* = 1, based on interaction analysis in logistic regression). Recovery time in clonidine groups of both patients with and without opium addiction was less than placebo ones which showed statistically significant difference (both *P* < 0.01, based on t-test). However the magnitude of the difference was higher in opium addicted than non-addicted patients (*P* = 0.04, based on interaction analysis in two way analysis of variance) as it is shown in the Figure 2 and Table 1 the recovery time difference between clonidine and placebo groups was around two minutes (108 vs. 110 min) in non-addicted patients where the difference was around five minutes (114 vs. 109 min) in opium addicted group.

**Table 1.** Baseline Characteristics of Patients

	Total	Control		*P* value a	Addict		*P* value a
		Placebo	Clonidine		Placebo	Clonidine	
**No.**	160	40	40		40	40	
**Age, y**							0.89
**Mean ± SD**	36.2 ± 12.8	32.9 ± 14.2	34.1 ± 12.2	0.68	38.8 ± 12.4	39.1 ± 11.3	
**Median (Range)**	34 (18 to 64)	27 (18 to 64)	33.5 (18 to 64)		38 (22 to 64)	37 (22 to 64)	
**Sex, M/F, Frequency (%)**	128/32 (80)	32/8 (80)	32/8 (80)	-	32/8 (80)	32/8 (80)	-
**Weight, kg**							0.06
**Mean ± SD**	71.6 ± 11	71.1 ± 13.1	73.9 ± 10.2	0.28	68.4 ± 9.9	72.8 ± 10.2	
**Median (Range)**	72 (48 to 110)	70 (48 to 110)	75.5 (52 to 100)		68 (51 to 90)	73 (55 to 100)	
**Duration of Operation, min**							0.51
**Mean ± SD**	110 ± 28	110 ± 27	108 ± 23	0.69	114 ± 30	109 ± 33	
**Median (Range)**	110 (40 to 180)	110 (50 to 170)	100 (60 to 170)		120 (40 to 180)	110 (40 to 180)	

^a^Based on t-test.

## 5. Discussion

The results from our study showed that clonidine as premedication can be an effective drug to decrease the incidence of shivering and recovery time after anesthesia for leg fracture operations not only in patients without addiction but also in opium addicted ones which their postoperative complications make a great challenge for anesthesiologists. Clonidine effectiveness upon preventing post anesthesia shivering has been reviewed before (17, 18). In one study, oral administration of clonidine 30 minutes before operation was assessed, their results showed that incidence of post anesthesia shivering was decreased but there was an increase in emergence time after operation (19). In another survey oral clonidine administration had better preventive effects on post anesthesia shivering than oral midazolam (20). We could not find any study related to the effect of clonidine on postoperative shivering in patients with opium addiction and we suggest that our study is a unique survey about this matter which showed that oral clonidine in patients with opium addiction can control the incidence of post op shivering. Clonidine premedication was found to decrease the recovery time after operation in both patients with and without opium addiction and even the magnitude of the difference between clonidine and placebo was higher in opium addicted than non-addicted patients. So, from our results we deducted that premedication effect of clonidine on post anesthesia recovery time in patients with opium addiction is better than patients without addiction and by considering the effectiveness of clonidine premedication on recovery time after anesthesia, recommendation about oral administration of clonidine as premedication in patients with opium addiction is reasonable. Some studies have shown that clonidine premedication does not delay recovery time after anesthesia (21, 22) but in other ones clonidine as premedication drug can delay arousal from anesthesia and extend the time of discharge from operating room (23, 24). Through our investigation we could not find any related article about clonidine effects on recovery time after anesthesia in opium abuser patients so, we suppose that our study upon the effectiveness of clonidine on recovery time after anesthesia in patients with opium addiction is a unique study on this matter. We could not have equal sexes of patients and the male ones were dominant in the study. So, it seems that we cannot generalize our results to both sexes. The other limitation was that we did not have any lab data about the level of opium in patients’ serum or urine and we relied on the history of patients about their addiction. It was due to an attempt to preserve patients’ rights and we did not intend to create any discomfort for patients to have any test about their addiction. In summary, the premedication of oral clonidine can be effective to decrease the incidence of shivering after operation in both patients with and without opium addiction and to decrease recovery time after anesthesia especially in patients with opium addiction.

## References

[1] Rajabizade G, Ramezani MA, Shakibi MR (2004). Prevalence of opium addiction in Iranian drivers 2001–2003.. J Med Sci..

[2] Hernandez M, Birnbach DJ, Van Zundert AA (2005). Anesthetic management of the illicit-substance-using patient.. Curr Opin Anaesthesiol..

[3] Anvari ZT, Afshar-Fereydouniyan N, Imani F, Sakhaei M, Alijani B, Mohseni M (2012). Effect of Clonidine Premedication on Blood Loss in Spine Surgery.. Anesth Pain..

[4] Ebneshahidi A, Mohseni M (2011). Premedication with oral clonidine decreases intraoperative bleeding and provides hemodynamic stability in cesarean section.. Anesth Pain..

[5] Gupta D, Srivastava S, Dubey RK, Prakash PS, Singh PK, Singh U (2011). Comparative evaluation of atenolol and clonidine premedication on cardiovascular response to nasal speculum insertion during trans-sphenoid surgery for resection of pituitary adenoma: A prospective, randomised, double-blind, controlled study.. Indian J Anaesth..

[6] Gupta K, Sharma D, Gupta PK (2011). Oral premedication with pregabalin or clonidine for hemodynamic stability during laryngoscopy and laparoscopic cholecystectomy: A comparative evaluation.. Saudi J Anaesth..

[7] Bergendahl H, Lonnqvist PA, Eksborg S (2005). Clonidine: an alternative to benzodiazepines for premedication in children.. Curr Opin Anaesthesiol..

[8] Bergendahl H, Lonnqvist PA, Eksborg S (2006). Clonidine in paediatric anaesthesia: review of the literature and comparison with benzodiazepines for premedication.. Acta Anaesthesiol Scand..

[9] Dabbagh A (2011). Clonidine: An old friend newly rediscovered.. Anesth Pain..

[10] Imani F, Rahimzadeh P, RezaFaiz SH (2011). Comparison the efficacy of adding clonidine, chloropromazine, promethazine and midazolam to morphine pumps in postoperative pain control of addicted patients.. Anesth Pain..

[11] Bryson EO (2011). The anesthetic implications of illicit opioid abuse.. Int Anesthesiol Clin..

[12] Najafi M, Sheikhvatan M (2012). Does Analgesic Effect of Opium Hamper the Adverse Effects of Severe Coronary Artery Disease on Quality of Life in Addicted Patients?. Anesth Pain..

[13] Bhattacharya P, Bhattacharya L, Jain R, Agarwal R (2003). Post anaesthesia shivering (PAS): A review article.. Indian J Anaesth..

[14] Sessler DI, Rubinstein EH, Moayeri A (1991). Physiologic responses to mild perianesthetic hypothermia in humans.. Anesthesiology..

[15] Crossley AW (1992). Peri-operative shivering.. Anaesthesia..

[16] Mathews S, Al Mulla A, Varghese PK, Radim K, Mumtaz S (2002). Postanaesthetic shivering--a new look at tramadol.. Anaesthesia..

[17] De Witte J, Sessler DI (2002). Perioperative shivering: physiology and pharmacology.. Anesthesiology..

[18] Zhao H, Ishiyama T, Oguchi T, Kumazawa T (2005). [Effects of clonidine and midazolam on postoperative shivering, nausea, and vomiting].. Masui..

[19] Mohammadi SS, Seyedi M (2007). Effects of Oral Clonidine in Preventing Postoperative Shivering After General Anesthesia.. Int J Pharm..

[20] Khullar Mahajan R, Singh I, Amar Parkash K (2012). Comparison of Oral Clonidine and Midazolam as Premedications in Children.. J Clinical Diagn Res..

[21] Bellaiche S, Bonnet F, Sperandio M, Lerouge P, Cannet G, Roujas F (1991). Clonidine does not delay recovery from anaesthesia.. Br J Anaesth..

[22] Paris A, Kaufmann M, Tonner PH, Renz P, Lemke T, Ledowski T (2009). Effects of clonidine and midazolam premedication on bispectral index and recovery after elective surgery.. Eur J Anaesthesiol..

[23] Ghai B, Ram J, Chauhan S, Wig J (2010). Effects of clonidine on recovery after sevoflurane anaesthesia in children undergoing cataract surgery.. Anaesth Intensive Care..

[24] Higuchi H, Adachi Y, Arimura S, Ogata M, Satoh T (2002). Oral clonidine premedication reduces the awakening concentration of propofol.. Anesth Analg..

